# Performance of gout definitions for genetic epidemiological studies: analysis of UK Biobank

**DOI:** 10.1186/s13075-017-1390-1

**Published:** 2017-08-09

**Authors:** Murray Cadzow, Tony R. Merriman, Nicola Dalbeth

**Affiliations:** 1 Department of Biochemistry, University of Otago, Dunedin, New Zealand; 2 Department of Medicine, Faculty of Medical and Health Sciences, University of Auckland, 85 Park Road, Grafton, Auckland, New Zealand

**Keywords:** Gout, Definition, Genetic, Epidemiology

## Abstract

**Background:**

Many different combinations of available data have been used to identify gout cases in large genetic studies. The aim of this study was to determine the performance of case definitions of gout using the limited items available in multipurpose cohorts for population-based genetic studies.

**Methods:**

This research was conducted using the UK Biobank Resource. Data, including genome-wide genotypes, were available for 105,421 European participants aged 40–69 years without kidney disease. Gout definitions and combinations of these definitions were identified from previous epidemiological studies. These definitions were tested for association with 30 urate-associated single-nucleotide polymorphisms (SNPs) by logistic regression, adjusted for age, sex, waist circumference, and ratio of waist circumference to height. Heritability estimates under an additive model were generated using GCTA version 1.26.0 and PLINK version 1.90b3.32 by partitioning the genome.

**Results:**

There were 2066 (1.96%) cases defined by *self-report of gout*, 1652 (1.57%) defined by *urate-lowering therapy (ULT) use*, 382 (0.36%) defined by *hospital diagnosis*, 1861 (1.76%) defined by *hospital diagnosis or gout-specific medications *and 2295 (2.18%) defined by *self-report of gout or ULT use*. Association with gout at experiment-wide significance (*P* < 0.0017) was observed for 13 SNPs with gout using the *self-report of gout or ULT use *definition, 12 SNPs using the *self-report* *of gout* definition, 11 SNPs using the *hospital diagnosis or gout-specific medication definition*, 10 SNPs using *ULT use* definition and 3 SNPs using *hospital diagnosis *definition. Heritability estimates ranged from 0.282 to 0.308 for all definitions except *hospital diagnosis *(0.236).

**Conclusions:**

Of the limited items available in multipurpose cohorts, the case definition of *self-report of gout or ULT use *has high sensitivity and precision for detecting association in genetic epidemiological studies of gout.

## Background

Accurate case definition is important for epidemiological studies. However, in multipurpose cohort studies frequently used for genetic epidemiological studies of gout, limited information is usually available for case definition. Many different combinations of available data have been used to identify gout cases in large genetic studies. For example, in the Global Urate Genetics Consortium study, the largest genome-wide association study (GWAS) of hyperuricaemia and gout reported to date, 15 different definitions of gout were used [1].

Population genetic studies frequently require large numbers of participants to achieve adequate statistical power, because common variants typically exert small effects on risk of disease. Within a study population, accurate case definition improves study power by maximising the number of true cases and minimising the number of falsely attributed disease-free control participants [2]. Consistent case definition is important for analyses that pool genetic data from different studies, as well as for those analyses that aim to replicate reported genetic associations.

Authors of a recent analysis of the Study for Updated Gout Classification Criteria (SUGAR), using synovial fluid confirmation of monosodium urate crystals as the gold standard for gout definition, reported that the definition of *self-report of gout or urate-lowering therapy (ULT) use* had the best test performance characteristics of existing definitions used in epidemiological studies [3]. The aim of the present study was to determine the performance of case definitions of gout using the limited items available in multipurpose cohorts, including *self-report of gout or ULT use*, for population-based genetic studies.

## Methods

This research was conducted using the UK Biobank Resource (approval number 12611) [4]. Data from the first tranche of UK Biobank genotyping and imputation data were used for this analysis (made publicly available in May 2015). Inclusion criteria were European ethnicity, age 40–69 years and genome-wide genotypes available. Exclusion criteria were self-reported sex mismatch with genetic sex, genotyping quality control failure, related individuals, either a primary or secondary hospital diagnosis of kidney disease (International Classification of Diseases, Tenth Revision (ICD-10), codes I12, I13, N00-N05, N07, N11, N14, N17–N19, Q61, N25.0, Z49, Z94.0, Z99.2), participants aged 70 years and over, and those with kidney disease, because these are risk factors for secondary gout.

Gout definitions and combinations of these definitions were identified from previous epidemiological studies [1, 3, 5]. *Self-report of gout *was defined by reporting of gout by the participant at the time of the study interview. *Hospital diagnosis of gout *was defined by either primary or secondary hospital discharge coding for gout (ICD-10 code M10, including sub-codes). *Use of ULT *required self-report of being on any of allopurinol, febuxostat or sulphinpyrazone and not having a hospital diagnosis of leukaemia or lymphoma (ICD-10 codes C81–C96). *Winnard-defined gout* was hospital diagnosis of gout or gout-specific medication (ULT or colchicine) as reported by Winnard et al. [5]. For participants who did not meet any gout definitions, further exclusion criteria were corticosteroid use, non-steroidal anti-inflammatory drug use or probenecid use.

UK Biobank samples had been genotyped using an Axiom array (820,967 markers; Affymetrix, Santa Clara, CA, USA) and imputed to approximately 73.3 million single-nucleotide polymorphisms (SNPs) using SHAPEIT3 and IMPUTE2 with a combined UK10K and 1000 Genomes reference panel. Logistic regression of SNPs against gout as the outcome was performed, adjusting for age, sex, waist circumference, and ratio of waist circumference to height. We analysed 30 urate-associated SNPs reported by Köttgen et al. in the large (>140,000 European participants) Global Urate Genetics Consortium GWAS [1]. Data were reported on the basis of number of SNPs detected at both genome-wide significance (*P* < 5 × 10^−8^) and experiment-wide significance (*P* < 0.0017). CIs for proportions were calculated using the Wilson score method and www.openepi.com [6]. Heritability estimates were compared using the formula h1-h2 (se = sqrt(se1^2 + se2 ^2)).

Heritability estimates under an additive model were generated using GCTA version 1.26.0 [7] and PLINK version 1.90b3.32 [8] by partitioning the genome. To reduce computational time, a smaller control cohort of 10,000 individuals was randomly generated from the UK Biobank and used for each set of cases. SNPs were filtered for deviation from Hardy-Weinberg equilibrium (*P* > 1 × 10^−6^) and minor allele frequency >0.01. A genetic relationship matrix was created for each chromosome, which was then used to calculate heritability assuming a prevalence of gout of 2% in the general population.

## Results

Data including genome-wide genotypes were available for 105,421 participants. Demographic and clinical data for the entire study group are shown in Table 1. Mean age was 56.87 years; 49.18% participants were male; and mean body mass index was 27.36 kg/m^2^.

**Table 1:** Participant characteristics (*n* = 105,421)

Variable	No. of subjects (%)
Age, years, mean (SD)	56.87 (7.92)
Male sex, *n* (%)	51,844 (49.2%)
Body mass index, kg/m^2^, mean (SD)	27.36 (4.73)
Diuretic use, *n* (%)	8939 (8.5%)
Waist circumference/height ratio, mean (SD)	0.536 (0.075)
Self-report of gout diagnosis, *n* (%)	2066 (2.0%)
Hospital diagnosis of gout, *n* (%)	382 (0.36%)
Allopurinol use, *n* (%)	1651 (1.7%)
Sulphinpyrazone use, *n* (%)	9 (0.01%)
Colchicine use, *n* (%)	63 (0.06%)
Febuxostat use, *n* (%)	0 (0%)

**Fig. 1:**
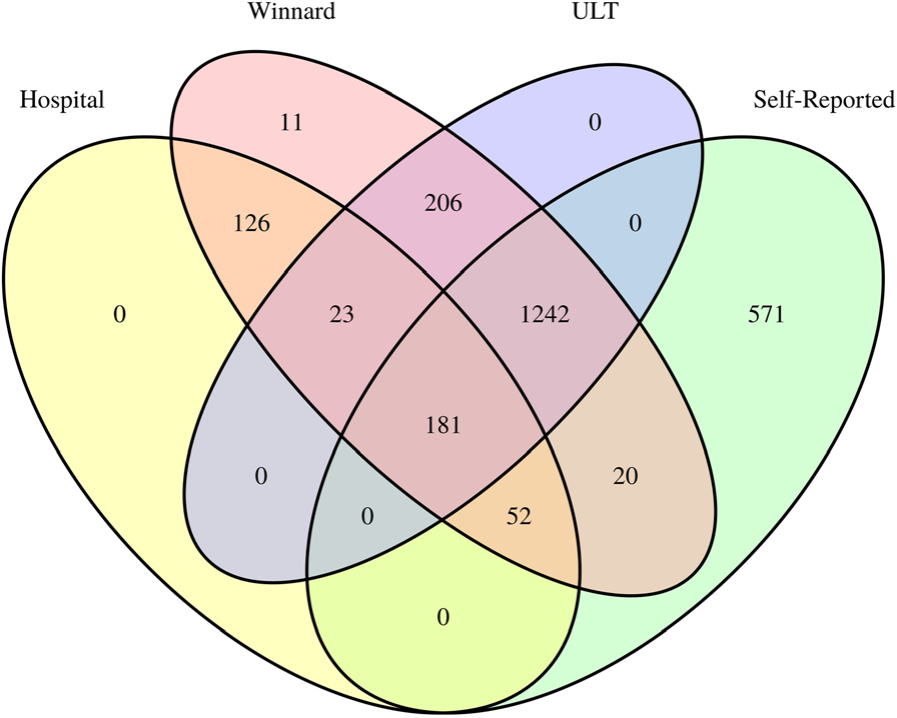
Number of patients fulfilling gout definitions. *ULT* Urate-lowering therapy

Figure 1 shows the number of cases identified by each gout definition. There was substantial overlap between most definitions. However, for those who met the *hospital diagnosis *criteria, 126 (33.0%) of 382 did not meet the *self-report of gout or ULT use *definition.

Table 2 shows the prevalence of gout identified by each gout definition in the entire study population and in men and women. The *hospital diagnosis *definition detected the fewest number of cases (*n* = 382, study population prevalence 0.36%). Definitions including *self-report of gout *detected significantly more cases than other definitions, with the definition of *self-report of gout or ULT use *detecting the highest number of cases (*n* = 2295, study population prevalence 2.18%).

**Table 2:** Number, prevalence (95% CI) of participants defined as gout cases

Definition	No. of subjects, prevalence (95% CI) in entire study population (*n* = 105,421)	No. of subjects, prevalence (95% CI) in male participants (*n* = 51,844)	No. of subjects, prevalence (95% CI) in female participants (*n* = 53,577)	Percentage (95% CI) of study population with gout using any definition (*n* = 2432)
*Self-report of gout diagnosis*	2066, 1.96% (1.88–2.05)	1921, 3.70% (3.55–3.87)	145, 0.27% (0.23–0.32)	84.95% (83.49–86.33)
*ULT use*	1652, 1.57% (1.49–1.64)	1529, 2.95% (2.81–3.10)	123, 0.23% (0.19–0.27)	67.93% (66.05–69.75)
*Hospital diagnosis*	382, 0.36% (0.33–0.40)	346, 3.29% (3.14–3.45)	36, 0.07% (0.05–0.09)	15.71% (14.32–17.21)
*Winnard definition* [5]^a^	1861, 1.76% (1.69–1.85)	1707, 3.29% (3.14–3.45)	154, 0.29% (0.25–0.34)	76.52% (74.80–78.16)
*Self-report of gout or ULT use*	2295, 2.18% (2.09–2.27)	2122, 4.09% (3.93–4.27)	173, 0.32% (0.28–0.37)	94.37% (93.38–95.22)

*ULT* Urate-lowering therapy

^a^Hospital diagnosis of gout or gout-specific medications

**Fig. 2:**
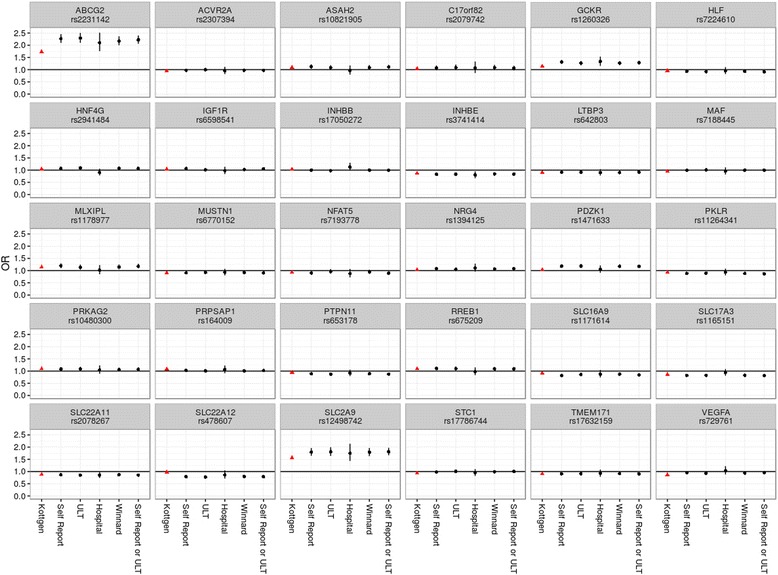
Plots showing ORs (95% CIs) for each single-nucleotide polymorphism according to tested gout case definitions. *Triangles* show ORs based on data from Köttgen et al. [1]. *ULT* Urate-lowering therapy

Analysis of the urate-associated SNPs described by Köttgen et al. [1] showed similar ORs for all gout definitions (Fig. 2, Table 3). However, the number of SNPs associated with gout at genome-wide or experiment-wide significance differed depending on gout case definition. Association with gout at genome-wide significance (*P* < 5 × 10^−8^) was observed for five SNPs (*ABCG2*, *SLC2A9*, *GCKR*, *SLC17A3* and *SLC22A12*) with gout defined by *self-report of gout or ULT use*, five SNPs (*ABCG2*, *SLC2A9*, *GCKR*, *SLC17A3* and *SLC22A12*) with gout defined by *self-report of gout*, four SNPs (*ABCG2*, *SLC2A9*, *GCKR* and *SLC17A3*) with gout defined by the *Winnard definition *[5], three SNPs (*ABCG2*, *SLC2A9* and *GCKR*) with gout defined by* ULT use *and two SNPs (*ABCG2* and *SLC2A9*) with gout defined by *hospital diagnosis*.

**Table 3:** ORs [95% CIs] and *P* values for hyperuricaemia single-nucleotide polymorphisms

	A1	A2	*Self-report* (*n* = 2066)	*ULT use* (*n* = 1652)	*Hospital diagnosis* (*n* = 382)	*Winnard* [5] *definition* (*n* = 1861)	*Self-report of gout or ULT use* (*n* = 2295)
Loci replicated by Köttgen							
*Rs2231142* (*ABCG2*)	T	G	2.10 [2.10–2.45], 2.38 × 10^−92^	2.29 [2.10–2.50], 4.22 × 10^−77^	2.10 [1.76–2.52], 3.57 × 10^−16^	2.18 [2.00–2.37], 4.47 × 10^−74^	2.22 [2.06–2.40], 1.93 × 10^−95^
*Rs12498742* (*SLC2A9*)	A	G	1.80 [1.65–1.97], 6.14 × 10^−39^	1.81 [1.64–2.00], 4.75 × 10^−32^	1.75 [1.43–2.14], 4.12 × 10^−8^	1.79 [1.63–1.97], 8.22 × 10^−35^	1.81 [1.67–1.97], 1.00 × 10^−43^
*Rs1260326* (*GCKR*)	T	C	1.31 [1.23–1.40], 2.22 × 10^−17^	1.26 [1.18–1.36], 9.30 × 10^−11^	1.33 [1.15–1.53], 0.00010	1.26 [1.18–1.35], 6.47 × 10^−12^	1.28 [1.21–1.36], 4.63 × 10^−16^
*Rs1165151* (*SLC17A3*)	T	G	0.83 [0.78–0.88], 5.36 × 10^−9^	0.83 [0.77–0.89], 1.47 × 10^−7^	0.94 [0.81–1.08], 0.38	0.83 [0.77–0.89], 4.43 × 10^−8^	0.82 [0.77–0.87], 7.87 × 10^−11^
*Rs478607* (*SLC22A12*)	A	G	0.79 [0.73–0.86], 2.32 × 10^−8^	0.78 [0.71–0.85], 5.42 × 10^−8^	0.87 [0.71–1.05], 0.14	0.79 [0.73–0.87], 2.18 × 10^−7^	0.79 [0.73–0.85], 2.00 × 10^−9^
*Rs1471633* (*PDZK1*)	A	C	1.18 [1.11–1.26], 2.60 × 10^−7^	1.19 [1.10–1.28], 2.25 × 10^−6^	1.05 [0.91–1.21], 0.50	1.18 [1.10–1.26], 1.54 × 10^−6^	1.17 [1.11–1.25], 1.86 × 10^−7^
*Rs3741414* (*INHBE*)	A	G	0.83 [0.76–0.89], 9.86 × 10^−7^	0.83 [0.76–0.91], 2.38 × 10^−5^	0.80 [0.67–0.95], 0.013	0.844 [0.78–0.92], 3.96 × 10^−5^	0.84 [0.78–0.90], 2.81 × 10^−6^
*Rs1171614* (*SLC16A9*)	T	C	0.82 [0.76–0.89], 1.11 × 10^−6^	0.86 [0.79–0.94], 0.00053	0.88 [0.74–1.05], 0.15	0.87 [0.81–0.95], 0.0012	0.84 [0.78–0.91], 4.78 × 10^−6^
*Rs2078267* (*SLC22A11*)	T	C	0.87 [0.82–0.93], 1.61 × 10^−5^	0.86 [0.80–0.92], 1.86 × 10^−5^	0.85 [0.74–0.98], 0.026	0.87 [0.81–0.93], 1.97 × 10^−5^	0.86 [0.81–0.91], 5.18 × 10^−7^
*Rs675209* (*RREB1*)	T	C	1.10 [1.03–1.18], 0.0056	1.10 [1.02–1.19], 0.017	0.98 [0.83–1.15], 0.80	1.09 [1.01–1.17], 0.024	1.09 [1.02–1.17], 0.0078
Loci reported by Köttgen							
*Rs11264341* (*PKLR*)	T	C	0.88 [0.83–0.94], 0.00013	0.89 [0.83–0.96], 0.0018	0.93 [0.81–1.08], 0.35	0.89 [0.83–0.95], 0.0004	0.87 [0.82–0.93], 1.18 × 10^−5^
*Rs17050272* (*INHBB*)	A	G	1.00 [0.94–1.07], 0.98	0.98 [0.91–1.05], 0.51	1.13 [0.97–1.30], 0.11	1.00 [0.93–1.07], 0.96	1.00 [0.94–1.06], 0.90
*Rs2307394* (*ACVR2A*)	T	C	0.97 [0.91–1.04], 0.40	1.00 [0.92–1.08], 0.96	0.96 [0.82–1.12], 0.57	0.97 [0.91–1.05], 0.47	0.97 [0.91–1.04], 0.39
*Rs6770152* (*MUSTN1*)	T	G	0.91 [0.85–0.97], 0.0042	0.93 [0.86–1.00], 0.04	0.93 [0.80–1.07], 0.31	0.92 [0.86–0.98], 0.015	0.91 [0.85–0.96], 0.0015
*Rs17632159* (*TMEM171*)	C	G	0.91 [0.85–0.98], 0.010	0.91 [0.84–0.99], 0.025	0.92 [0.79–1.08], 0.32	0.92 [0.85–0.99], 0.025	0.91 [0.85–0.97], 0.0064
*Rs729761* (*VEGFA*)	T	G	0.95 [0.89–1.02], 0.19	0.93 [0.86–1.01], 0.079	1.04 [0.88–1.22], 0.67	0.94 [0.87–1.01], 0.10	0.96 [0.89–1.03], 0.21
*Rs1178977* (*MLXIPL*)	A	G	1.19 [1.10–1.30], 3.49 × 10^−5^	1.14 [1.04–1.25], 0.0066	1.02 [0.85–1.22], 0.81	1.15 [1.05–1.25], 0.0020	1.18 [1.09–1.28], 3.79 × 10^−5^
*Rs10480300* (*PRKAG2*)	T	C	1.08 [1.01–1.16], 0.026	1.09 [1.01–1.18], 0.032	1.05 [0.89–1.23], 0.57	1.06 [0.99–1.14], 0.119	1.08 [1.01–1.15], 0.028
*Rs17786744* (*STC1*)	A	G	0.98 [0.92–1.05], 0.53	1.01 [0.94–1.08], 0.86	0.95 [0.82–0.10], 0.49	0.99 [0.93–1.06], 0.82	1.00 [0.94–1.06], 0.99
*Rs2941484* (*HNF4G*)	T	C	1.07 [1.00–1.14], 0.037	1.09 [1.01–1.17], 0.023	0.91 [0.78–1.06], 0.22	1.08 [1.00–1.15], 0.040	1.07 [1.01–1.14], 0.029
*Rs10821905* (*ASAH2*)	A	G	1.12 [1.04–1.22], 0.0049	1.08 [0.99–1.18], 0.10	0.97 [0.80–1.17], 0.73	1.09 [1.00–1.19], 0.051	1.11 [1.03–1.20], 0.0080
*Rs642803* (*LTBP3*)	T	C	0.92 [0.86–0.98], 0.011	0.92 [0.85–0.98], 0.016	0.90 [0.78–1.04], 0.16	0.90 [0.84–0.96], 0.0025	0.92 [0.87–0.89], 0.0090
*Rs653178* (*PTPN11*)	T	C	0.89 [0.83–0.94], 0.00015	0.87 [0.81–0.93], 0.00012	0.92 [0.79–1.06], 0.24	0.89 [0.83–0.95], 0.00061	0.88 [0.83–0.93], 3.11 × 10^−5^
*Rs1394125* (*NRG4*)	A	G	1.08 [1.01–1.15], 0.026	1.05 [0.98–1.30], 0.19	1.11 [0.96–1.29], 0.17	1.07 [0.99–1.14], 0.074	1.07 [1.01–1.14], 0.027
*Rs6598541* (*IGF1R*)	A	G	1.07 [1.00–1.40], 0.059	1.01 [0.94–1.09], 0.78	0.98 [0.84–1.14], 0.75	1.02 [0.95–1.10], 0.55	1.05 [0.99–1.12], 0.12
*Rs7193778* (*NFAT5*)	T	C	0.90 [0.83–0.98], 0.021	0.96 [0.87–1.06], 0.43	0.88 [0.72–1.07], 0.19	0.95 [0.86–1.04], 0.25	0.90 [0.83–0.97], 0.0087
*Rs7188445* (*MAF*)	A	G	0.99 [0.93–1.06], 0.87	1.01 [0.94–1.09], 0.82	0.96 [0.82–1.11], 0.56	1.00 [0.93–1.07], 0.95	1.00 [0.94–1.06], 0.94
*Rs7224610* (*HLF*)	A	C	0.93 [0.87–0.99], 0.022	0.92 [0.85–0.99], 0.022	0.95 [0.82–1.10], 0.51	0.94 [0.87–1.00], 0.058	0.92 [0.86–0.98], 0.0061
*Rs2079742* (*C17ORF82*)	T	C	1.07 [0.97–1.17], 0.18	1.09 [0.97–1.21], 0.14	1.07 [0.86–1.33], 0.54	1.08 [0.98–1.20], 0.13	1.06 [0.97–1.16], 0.23
*Rs164009* (*PRPSAP1*)	A	G	1.03 [0.96–1.09], 0.45	1.01 [0.94–1.09], 0.83	1.06 [0.91–1.23], 0.45	1.01 [0.94–1.08], 0.80	1.02 [0.96–1.09], 0.47
Number of loci associated with gout at genome wide significance			5	3	2	4	5
Number of loci associated with gout at experiment wide significance			12	10	3	11	13

*ULT* Urate-lowering therapy

Data are adjusted by age, sex, waist circumference, and waist-to-height ratio. Experiment-wide significance is defined as *P* < 0.0017. Hyperuricaemia single-nucleotide polymorphisms are as described by Köttgen et al. [1] using different gout definitions

Association with gout at experiment-wide significance (*P* < 0.0017) was observed for 13 SNPs with gout defined by *self-report of gout or ULT use*, for 12 SNPs with gout defined by *self-report*, for 11 SNPs with gout defined by the *Winnard definition*, for 10 SNPs with gout defined by *ULT use*, and for 3 SNPs with gout defined by *hospital diagnosis *(Table 3). The heritability estimates (i.e., proportion of variance in gout explained by common inherited genetic variants under an additive model of inheritance) were 0.289 (0.034) for the *self-report of gout or ULT use *definition, 0.283 (0.036) for the *self-report of gout* definition, 0.282 (0.040) for the Winnard definition, 0.308 (0.044) for the *ULT use* definition and 0.236 (0.160) for the *hospital diagnosis *definition. There were no significant differences between the heritability estimates.

## Discussion

Accurate and consistent phenotyping of cases and disease-free control participants is important to maximise study power and reduce the risk of misclassification bias in genetic association studies. Consistent definitions of disease phenotypes are also important for replication of genetic associations in different cohorts [9]. In this analysis of UK Biobank data, the definition of *self-report of gout or ULT use* detected the highest number of gout cases and had greatest precision for genetic association analysis.

Our findings are consistent with a recent analysis of the SUGAR cohort that used synovial fluid confirmation of monosodium urate crystals as the gold standard for gout definition [3]. The SUGAR analysis reported that the definition of *self-report of gout or ULT use *had the best test performance characteristics of existing definitions, with sensitivity of 82% and specificity of 72%. Collectively, these data support the use of the *self-report of gout or ULT use *definition for use in epidemiological studies when more detailed gout-specific clinical data are not available.

The different definitions of gout used in this study may reflect different disease presentations or patient populations. Although not all patients were captured by any definition, there was substantial overlap between most definitions. The definition of *hospital diagnosis *is very restrictive and is unlikely to capture most people with gout. Of note, 126 (33.0%) of 382 of those who met the *hospital diagnosis *criteria did not meet the *self-report of gout or ULT use *definition. There may be several reasons for this. First, the hospitalised population may have a different disease presentation from that of those identified in the community through self-report or ULT use. Furthermore, a diagnosis of gout made during a hospital admission may subsequently be revised to a different diagnosis, and the ascertainment methodology does not take this into account. Compared with the case definition of *self-report of gout or ULT use*, the *Winnard definition *led to a lower estimated prevalence of gout and also had lower precision for genetic association analysis. Therefore, when self-report information is available, we recommend the definition of *self-report of gout or ULT use*.

For all definitions tested, *ABCG2* and *SLC2A9* were associated with gout at genome-wide significance. These genes encode proteins that regulate uric acid transport within the gut and proximal renal tubule, respectively. The large effect sizes observed in this study are reminiscent of their dominant effect sizes in GWAS of control of serum urate levels [1], consistent with the central role of these two genes in regulating serum urate and gout risk. As part of evaluating the various definitions, we also calculated heritability estimates of gout, with the proportion of age-, sex- and body composition-adjusted variance explained by all common SNPs to be 0.282–0.308 (excluding the hospital definition). Previously, Köttgen et al. [1], also using GCTA software, had estimated a range of genome-wide heritability estimates of 0.27–0.41 for age- and sex-adjusted serum urate levels, depending on the individual sample sets analysed. The estimates of variance explained in serum urate and gout by common genetic variants in the European sample sets are comparable, suggesting that the common genetic variant-mediated heritabilities of serum urate levels and gout are similar. Clearly, environmental factors also contribute to the risk of gout, such as dietary exposures and medications. The heritability estimates use information from common SNPs under the assumption of additive contributions. Therefore, the estimates will not include the contribution of non-additive gene-by-gene and gene-by-environment interactions, rare genetic variants and copy number variations.

We acknowledge that our study has limitations. The analysis was restricted to European participants, and our genetic association results may not be generalisable to non-European populations. Furthermore, a definition that includes ULT may be less specific for gout if the study population is recruited from countries in which ULT is recommended for treatment of asymptomatic hyperuricaemia. A diagnostic gold standard was not available in this study, and therefore it is not possible to determine the false-positive or false-negative rates using this dataset. Disease validation was based on the genotype data available in this cohort, and gout was inferred on the basis of known genetic associations with hyperuricaemia and gout. The strength of association observed in this study population may not reflect findings in the general UK population; risk factors for secondary gout (age ≥70 years and kidney disease) were exclusion criteria. The study findings also are not applicable to studies in which researchers do not collect information about self-report of gout or gout medication use. Our study’s strengths include the large sample size with consistent data collection. The comprehensive data collection, including patient interviews, hospitalisation records and medication information, allowed us to compare a number of different case definitions within a single study.

## Conclusions

The case definition of *self-report of gout or ULT use *has high precision for detecting association in genetic epidemiological studies of gout. When these variables are available within multi-purpose cohorts, the consistent use of this case definition should reduce the risk of misclassification bias and improve study power.

## Abbreviations

GWAS: Genome-wide association study
ICD-10: International Classification of Diseases, Tenth Revision
SNP: Single-nucleotide polymorphism
SUGAR: Study for Updated Gout Classification Criteria
ULT: Urate-lowering therapy
